# Clinicopathological comparison between acute gastrointestinal-graft-versus-host disease and infectious colitis in patients after hematopoietic stem cell transplantation

**DOI:** 10.1371/journal.pone.0200627

**Published:** 2018-07-30

**Authors:** Sae Ohwada, Tomoya Iida, Daisuke Hirayama, Gota Sudo, Toshiyuki Kubo, Masanori Nojima, Kentaro Yamashita, Hiroo Yamano, Hiroshi Nakase

**Affiliations:** 1 Department of Gastroenterology and Hepatology, Sapporo Medical University School of Medicine, Sapporo, Japan; 2 Center for Translational Research, The Institute of Medical Science Hospital, The University of Tokyo, Tokyo, Japan; University of Kentucky, UNITED STATES

## Abstract

The aim of this study is to elucidate the differences of the clinicopathological characteristics between acute gastrointestinal (GI)-graft-versus-host disease (GVHD) and infectious colitis (IC) after hematopoietic stem cell transplantation (HSCT). Of the 282 patients who underwent HSCT at our institution between January 1991 and December 2015, we could investigate 182 patients in detail. Of the 182 patients, we selected those who underwent colonoscopy and were diagnosed with acute GI-GVHD or IC after HSCT. Patients’ backgrounds, colonoscopic findings, and pathological findings were retrospectively analyzed. There were 30 patients who had colonoscopy performed and diagnosed with acute GI-GVHD or IC after HSCT. Of the 30 patients, 20 had acute GI-GVHD and 10 had IC. All the cases of acute GI-GVHD were diagnosed by endoscopic biopsy and 4 of the IC patients had *Clostridium difficile* associated colitis. In the IC group, the period from the transplantation up to diagnosis was significantly shorter than acute GI-GVHD group (10.0 days vs. 43.2 days, p = 0.03). In the acute GI-GVHD group, tortoiseshell-like mucosal patterns were significantly more common than the IC group (70% vs. 0%, p < 0.001). Furthermore, there were some cases presenting normal mucosal appearance despite the diagnosis with acute GI-GVHD by pathological findings. Clinically, we should consider IC when abdominal symptoms appeared in the early period after HSCT. Endoscopically, tortoiseshell-like mucosal pattern was a characteristic feature of acute GI-GVHD. In addition, it is essential to perform mucosal biopsy for diagnose of acute GI-GVHD even in patients showing the normal mucosal appearance.

## Introduction

Hematopoietic stem cell transplantation (HSCT), also known as bone marrow transplantation (BMT), peripheral blood stem cell transplantation (PBSCT), and cord blood transplantation (CBT), involves the intravenous infusion of hematopoietic stem cells collected from the bone marrow, peripheral blood, and umbilical cord blood, respectively. HSCT has been performed with increasing frequency for the common treatment of numerous malignant and nonmalignant diseases, such as leukemia, malignant lymphoma (ML), multiple myeloma (MM), myelodysplastic syndromes (MDS), aplastic anemia (AA), and other disorders of the hematopoietic system. The development of acute and chronic graft-versus-host disease (GVHD), which can lead to life-threatening processes [1], is one of the significant complications after HSCT.

Acute GVHD is defined as GVHD that occurs within the first 100 days of allogeneic (allo)-HSCT. Acute GVHD is a major cause of morbidity and mortality in patients who undergo HSCT [2]. Although acute GVHD mainly affects the skin, the gastrointestinal (GI) tract is one of the most sensitive organ systems that is affected by GVHD [2]. GI-GVHD results from damage to the recipient’s GI epithelium caused by the donor’s lymphocytes. Acute GI-GVHD was reported in 30%–70% of patients who underwent HSCT [3–6].

Patients with acute GI-GVHD usually present with frequent watery diarrhea, abdominal pain, and hematochezia; similar complaints are also seen in patients with HSCT accompanied with infectious colitis (IC). In the pre- and early post-HSCT phases, patients are at a high risk for developing infections, which are caused by bacteria (*Clostridium difficile* [*CD*], *Pseudomonas*, and *Klebsiella* species), fungi (*Aspergillus* and *Candida* species), viruses (cytomegalovirus [CMV] and herpes simplex virus), and parasites. In particular, *CD* and CMV infections are the most commonly occurring GI infections after HSCT. The treatment of acute GI-GVHD is quite different from IC. Therefore, it is important to distinguish acute GI-GVHD from IC in patients with HSCT for initiating optimal treatments.

Several studies have demonstrated the clinicopathological characteristics of GI-GVHD [7–12]. Nevertheless, no studies were found directly compare the differences between acute GI-GVHD and IC except for a study using barium [13]. Our study aimed to elucidate the clinicopathological differences between acute GI-GVHD and IC in patients with HSCT.

## Materials and methods

### Ethical considerations

This study obtained ethical approval from the institutional review board of Sapporo Medical University. The data used in this study were de-identified and released to the public for research purposes. Therefore, the institutional review board of Sapporo Medical University waived informed consent from the enrolled patients. This study was conducted in accordance with the Declaration of Helsinki.

### Study protocol

A total of 282 patients underwent HSCT at our institution between January 1991 and December 2015. Of the 282 patients, 182 patients have been investigated in detail. Of the 182 patients, we selected those who underwent colonoscopy (CS) and were diagnosed with acute GI-GVHD or IC after HSCT. The patients who did not undergo CS, those who had a history of severe GI disease before the transplantation, and those who coexisted with GI-GVHD and IC were excluded.

**Fig 1:**
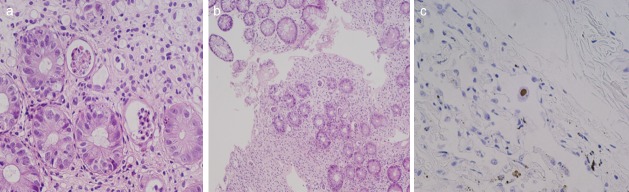
Pathological findings. (a) Crypt cell apoptosis in patients with acute GI-GVHD, (b) Ductal atrophy in patients with acute GI-GVHD, (c) Inclusion body in patients with CMV infection.

To diagnose acute GI-GVHD, an endoscopic biopsy was performed in all the cases, which was based on the presence of crypt cell degeneration or crypt cell apoptosis, with or without crypt loss (Fig 1A) [8,14,15]. If such findings were not observed, other histological findings, such as ductal atrophy (Fig 1B) or development of crypt abscesses, were included for definitive diagnosis. In the present study, biopsy specimens were stained using hematoxylin and eosin and evaluated according to the criteria for the histological diagnosis of acute GVHD. In contrast, to diagnose IC, fecal culture, *CD* toxin testing, CMV antigenemia, and endoscopic biopsy were performed. Endoscopic biopsy was performed to diagnose CMV enteritis (Fig 1C) and to exclude GI-GVHD.

Distinguishing thrombotic microangiopathy (TMA) from acute GI-GVHD and IC is required. TMA was excluded based on the laboratory findings and pathological findings, which pointed to the presence of hyaline thrombi in the capillaries, or fibrinoid necroses of the terminal arterioles of the intestine.

### Endoscopic procedure

CS was performed to observe the entire colon and terminal ileum. However, in patients with poor condition or cases involving difficult colonoscopy (difficulty in intubation), the endoscopic observation was limited to the site where the scope could reach. The biopsy samples were collected from the normal mucosa and the intestinal mucosa, which demonstrated endoscopic features, such as redness, edema, erosion, and ulceration. CS was performed using an Olympus colonoscope (CF-Q240/260AI, CF-Q260ZI, CF-HQ290ZI, PCF-260AI; Olympus Medical Systems Co., Tokyo, Japan) with indigo carmine dye contrast, and narrow band imaging, with or without magnifying observation, by experienced endoscopists. Sedatives were administered according to the patients’ conditions.

### Examination items

Patients’ characteristics (mean age, sex, type of HSCT, mean days from HSCT to diagnosis [from HSCT to the day performed CS or stool test], mean frequency of diarrhea at the onset of enteritis), endoscopic and histopathological findings, and clinical courses were reviewed from medical charts and films, retrospectively.

### Statistical analysis

The significant differences in the variable characteristics were investigated using Fisher’s exact test for categorical variables and a Student’s t-test for continuous variables. EZR [16], version 1.32, was used to record and analyze the data. The differences were considered significant when the two-sided p-value was <0.05.

## Results

The backgrounds of the 182 patients are shown in Table 1. The mean age of the patients was 52.8 years (2–69 years). The primary diseases that warranted a transplantation included acute myelogenous leukemia (n = 35), acute lymphoblastic leukemia (n = 6), chronic myelogenous leukemia (n = 1), ML (n = 63), MM (n = 52), MDS (n = 14), AA (n = 2), and others (n = 9). BMT (n = 41), PBSCT (n = 128), and CBT (n = 13) were the types of transplantation included.

**Table 1:** Backgrounds of 182 patients who underwent hematopoietic stem cell transplantation.

Mean Age (years, range)		52.8 (2–69)
Sex (M: F)		102: 80
Type of primary disease	AML	35
	ALL	6
	CML	1
	CLL	0
	ML	63
	MM	52
	MDS	14
	AA	2
	Others	9
Type of HSCT	BMT	41
	PBSCT	128
	CBT	13
	Others	0

HSCT: hematopoietic stem cell transplantation

AML: acute myelogenous leukemia, ALL: acute lymphoblastic leukemia

CML: chronic myelogenous leukemia, CLL: chronic lymphoblastic leukemia

ML: malignant lymphoma, MM: multiple myeloma

MDS: myelodysplastic syndromes, AA: aplastic anemia

BMT: bone marrow transplantation

PBSCT: peripheral blood stem cell transplantation

CBT: cord blood transplantation

**Fig 2:**
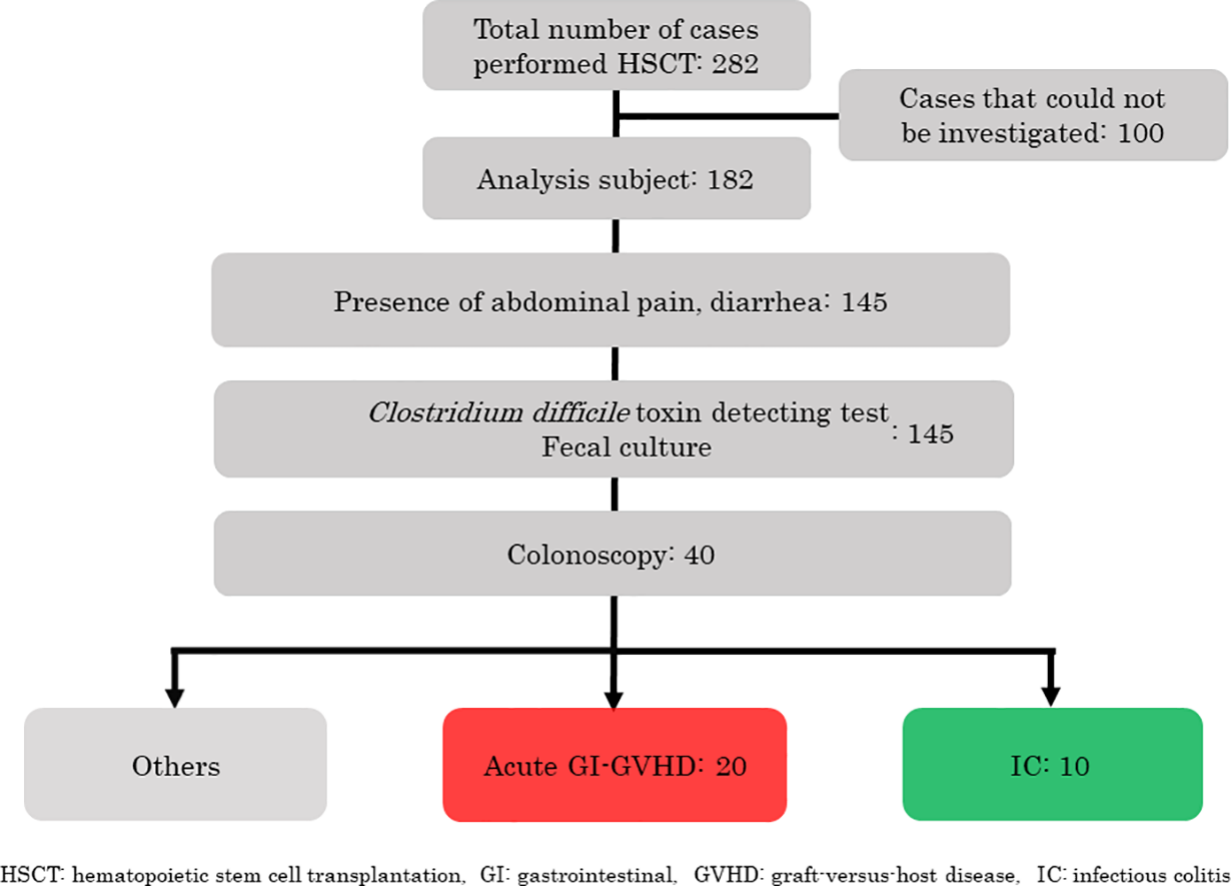
Analysis subject.

Subject analysis is shown in Fig 2. Of the 182 patients, 145 patients exhibited GI symptoms, including abdominal pain and diarrhea. *CD* toxin testing and fecal cultures were performed in all the patients. In addition, CS was performed in 40 of these patients. Of the 40 patients, acute GI-GVHD was observed in 20 patients, and IC was observed in 10 patients (*CD* [n = 4], *Pseudomonas aeruginosa* [n = 3], *Klebsiella oxytoca* [n = 1], *Trichosporon asahii* [n = 1], and CMV [n = 1]). Remaining 10 patients were performed not only colonoscopy but also esophagogastroduodenoscopy (EGD). Then, they were not diagnosed with GI-GVHD or IC by the result of endoscopic histologic examination and stool culture. It was considered that the abdominal symptoms of these 10 patients were associated with pretreatments prior to HSCT. No patients with TMA were observed.

Table 2 shows the characteristics of patients in the acute GI-GVHD and IC groups. Significant differences in types of transplantation and the mean number of days from HSCT were observed between the GI-GVHD and IC group. In contrast, no significant differences were observed in the mean age, sex, and the mean frequency of diarrhea at the onset of enteritis between the two groups.

**Table 2:** Backgrounds of patients with acute gastrointestinal graft-versus-host disease or infectious colitis.

	Acute GI-GVHD (n = 20)	IC (n = 10)	p value
Mean Age (years, range)	44.1 (2–67)	52.0 (26–64)	0.36
Sex (M: F)	13: 7	7:3	1
Type of HSCT (BMT/PBSCT/CBT)	14/0/6	3/7/0	<0.01
Mean frequency of diarrhea (/day)	5.9	5.4	0.86
Mean Days from HSCT to diagnosis (days)	43.2	10.0	0.03

GI: gastrointestinal, GVHD: graft-versus-host disease, IC: infectious colitis M: male, F: female, HSCT: hematopoietic stem cell transplantation, BMT: bone marrow transplantation, PBSCT: peripheral blood stem cell transplantation, CBT: cord blood transplantation

**Fig 3:**
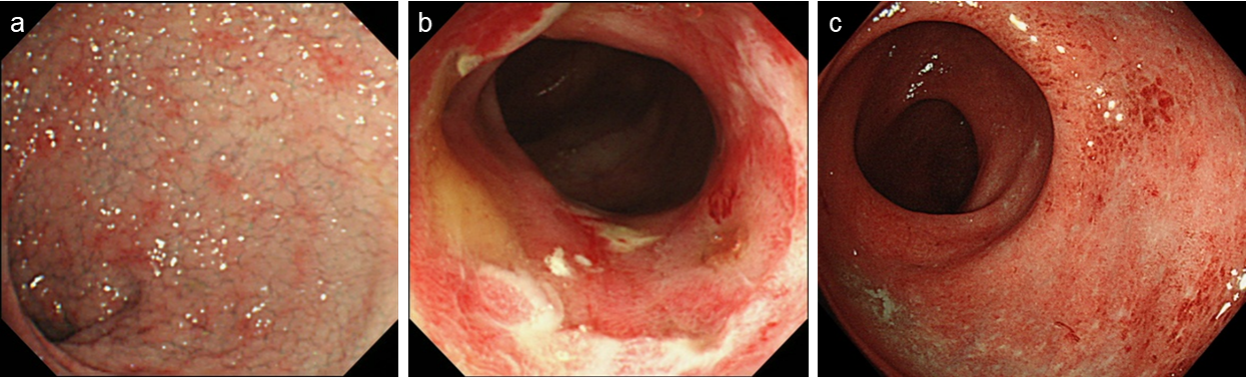
Endoscopic findings. (a) tortoiseshell-like mucosal pattern in patients with acute GI-GVHD, (b) erosion/ulceration in patients with acute GI-GVHD, (c) redness in patients with infectious colitis.

The endoscopic findings of acute GI-GVHD and IC groups are shown in Table 3. GI-GVHD patients had a high frequency of tortoiseshell-like mucosal pattern (aggregated granular edematous lesion) (70%: 14/20) (Fig 3A), while these endoscopic features were less common in patients with IC (0%: 0/10) with statistical significance (*p* <0.001). In acute GI-GVHD group, tortoiseshell-like mucosal pattern was found in 10, 4, and 2 of 14 patients (including duplicate) in rectosigmoid, left-side, and right-side colon, respectively. No significant differences in erosion/ulceration (Fig 3B), redness (Fig 3C), edema, and normal appearance were observed between the two groups. However, two of 20 patients with GI-GVHD (10%) showed normal mucosal appearance.

**Table 3:** Lower gastrointestinal endoscopic findings in patients with acute gastrointestinal graft-versus-host disease or infectious colitis.

	Acute GI-GVHD (n = 20)	IC (n = 10)	p value
Tortoiseshell-like pattern	70%	0%	<0.001
Erosion/Ulceration	30%	0%	0.07
Redness	65%	60%	1
Edema	40%	80%	0.42
Normal	10%	0%	0.54

GI: gastrointestinal, GVHD: graft-versus-host disease, IC: infectious colitis

The pathological findings of acute GI-GVHD and IC groups are shown in Table 4. No specific pathological findings for diagnosis of IC were identified.

**Table 4:** Pathological findings of the biopsies during lower gastrointestinal endoscopy in patients with acute gastrointestinal graft-versus-host disease or infectious colitis.

	Acute GI-GVHD (n = 20)	IC (n = 8)	p value
Crypt cell apoptosis	75%	0%	0.005
Ductal atrophy	35%	0%	0.16
Crypt abscess	10%	25%	0.41
Inflammatory cell infiltration	40%	75%	0.20
Inclusion body	0%	20%	0.20

GI: gastrointestinal, GVHD: graft-versus-host disease, IC: infectious colitis

## Discussion

The aim of this study is to evaluate the differences in the clinicopathological characteristics between patients with acute GI-GVHD and with IC. Three important points were found: (1) abdominal symptoms appeared in HSCT patients with IC significantly earlier than those with acute GI-GVHD; (2) a tortoise shell-like mucosal pattern was a characteristic endoscopic feature of acute GI-GVHD; and (3) sufficient amount of biopsy specimens should be taken from the GI tract because some patients with acute GI-GVHD had endoscopically normal mucosal appearance.

No reports were found comparing the difference of the mean time to diagnosis between acute GI-GVHD and IC. Wild et al. [17] showed that the median time to diagnosis in 169 patients with acute GI-GVHD after allo-HSCT was 84 days. Holmberg et al. [18] reported that the mean time to diagnosis in 181 patients with acute GI-GVHD after HSCT was 42 days. In contrast, Alonso et al. [19] showed that that a median time to *CD* infection (CDI) from auto-HSCT was 6.5 days. According to Willems et al. [20], the median time to CDI from HSCT was 25 days. Taken together, previous reports and current data suggest that IC, including CDI, are likely to occur relatively earlier after HSCT in comparison with GI-GVHD. Possibly, the daily use of antibiotics and significant reduction of immune response against various pathogens because of pre-transplant treatments and HSCT might be involved in the early onset.

In the present study, a tortoiseshell-like mucosal pattern was a characteristic endoscopic finding. Fig 3A shows an edematous mucosa in which the mucosal surface seems to be finely cracked and colonic pits are exaggerated, which was more clearly observed using chromoendoscopy with indigo carmine dye spraying. Tortoiseshell-like mucosal pattern appeared because of histopathologically maintained surface structure and the presence of remarkable edema in the lamina propria, without inflammatory cell infiltration. Hiejima et al. [12] reported that acute GI-GVHD was significantly frequently associated with short blunt villi in the duodenum, variable defect villi and short blunt villi in the ileum, and edema, erosion, and tortoiseshell-like mucosa in the colon. Consistent with his report, tortoiseshell-like mucosal pattern was found to be one of the endoscopic characteristics of adult GI-GVHD. It was important that the present study supported it.

Ten percent of the patients diagnosed with acute GI-GVHD showed endoscopically normal mucosal appearance. In some cases, skin biopsy can be used for GVHD diagnosis; however, biopsy from GI can also be used because its diagnostic rate is higher (58%) than that of the skin biopsy (46%) [21]. A positive association between the endoscopic grading and the histological grading of GI-GVHD has been reported previously [22,23]. Cox et al. [24] reported that the rate of observed normal endoscopic appearance was 21% in patients with histologically confirmed acute GI-GVHD. Aslanian et al. [25] showed that 60% and 46% of patients with GI-GVHD had endoscopically normal appearance through EGD and CS, respectively. In addition, a report on pediatric patients after HSCT revealed that the most common endoscopic finding was normal mucosa, seen in 25%, 57%, and 50% of gastric, duodenal, and rectosigmoid examinations, respectively [26]. Therefore, if acute GI-GVHD is suspected after HSCT, GI biopsy should be actively performed even in patients with no abnormal endoscopic findings.

The limitations of this study included the fact that (1) the proportion of patients with acute GI-GVHD was lower than those observed in other studies [3–6], (2) the proportion of patients with CMV infection was lower than those observed in other studies [27–30], (3) we could not find out useful markers to distinguish between acute GI-GVHD and IC, and (4) a single-center retrospective study was conducted with a small sample size. The lower diagnostic rate of acute GI-GVHD in this study may have been affected by the fact that (i) the enrolled patients were limited to the cases diagnosed with acute GI-GVHD by CS because the focus was on the endoscopic and pathological findings in the lower GI and (ii) the rate of observing the entire colon including the terminal ileum was slightly low (60%) in this study. The present study showed tortoiseshell-like mucosal pattern was found more in distal colon. However, Thompson et al. [31] demonstrated that the positive rate of endoscopic biopsy using colonoscopy with ileal intubation was higher than using only colonoscopy (95% vs. 82%). Peyer’s patch is the essential site in initiating acute and lethal graft-versus-host reaction [32]. Therefore, careful observation of the entire colon including the terminal ileum is required to diagnose GI-GVHD. In addition, the lower diagnostic rate of CMV infection in this study could be attributable to the fact that our patients did not commonly undergo PCR examination for CMV in the blood or colonic tissue.

## Conclusions

This study was conducted to evaluate the differences of the clinicopathological characteristics between patients with acute GI-GVHD and with IC. Clinically, the presence of IC should be considered when abdominal symptoms appeared in the early period after HSCT. Endoscopically, tortoiseshell-like mucosal pattern is a characteristic feature of acute GI-GVHD. Biopsy should be performed even in patients showing normal mucosal appearance to diagnose acute GI-GVHD. Future studies utilizing prospective and larger sample sizes are therefore necessary to validate the results of the present study and to identify useful markers to distinguish between acute GI-GVHD and IC.

### Compliance with ethical standards

The authors declare no conflict of interest. This work was supported in part by Health and Labour Sciences Research Grants for research on intractable diseases from the Ministry of Health, Labour and Welfare of Japan (Investigation and Research for intractable Inflammatory Bowel Disease) and Japan Society for the Promotion of Science (JSPS) KAKENHI Grant Number JP17J02428 (to TI). The funders of the study had no role in the study design, data collection, data analysis, data interpretation, or writing of the report.
